# Once-nightly sodium oxybate (FT218) demonstrated improvement of symptoms in a phase 3 randomized clinical trial in patients with narcolepsy

**DOI:** 10.1093/sleep/zsab200

**Published:** 2021-08-06

**Authors:** Clete A Kushida, Colin M Shapiro, Thomas Roth, Michael J Thorpy, Bruce C Corser, Akinyemi O Ajayi, Russell Rosenberg, Asim Roy, David Seiden, Jordan Dubow, Yves Dauvilliers

**Affiliations:** Department of Psychiatry and Behavioral Sciences, Stanford University Medical Center, Redwood City, CA, USA; University of Toronto, Toronto, ON, Canada; Sleep Disorders and Research Center, Henry Ford Health System, Detroit, MI, USA; Department of Neurology, Montefiore Medical Center, New York, NY, USA; Sleep Management Institute, Cincinnati, OH, USA; Florida Pediatric Research Institute, Winter Park, FL, USA; Neurotrials Research, Inc., Atlanta, GA, USA; Ohio Sleep Medicine and Neuroscience Institute, Dublin, OH, USA; Avadel Pharmaceuticals, Chesterfield, MO, USA; Avadel Pharmaceuticals, Chesterfield, MO, USA; National Reference Centre for Orphan Diseases, Narcolepsy, Idiopathic Hypersomnia, Sleep Unit, Department of Neurology, Gui-de-Chauliac Hospital, CHU Montpellier, Univ Montpellier, INM INSERM, Montpellier, France

**Keywords:** narcolepsy, sodium oxybate, once-nightly, NT1/NT2, safety, modified release

## Abstract

**Study Objectives:**

To assess the efficacy and safety of FT218, a novel once-nightly formulation of sodium oxybate (ON-SXB), in patients with narcolepsy in the phase 3 REST-ON trial.

**Methods:**

Narcolepsy patients aged ≥16 years were randomized 1:1 to uptitration of ON-SXB (4.5, 6, 7.5, and 9 g) or placebo. Three coprimary endpoints were change from baseline in mean sleep latency on the Maintenance of Wakefulness Test, Clinical Global Impression-Improvement rating, and weekly cataplexy attacks at 9, 7.5, and 6 g. Secondary endpoints included change from baseline on the Epworth Sleepiness Scale. Safety included adverse drug reactions and clinical laboratory assessments.

**Results:**

In total, 222 patients were randomized; 212 received ≥1 dose of ON-SXB (*n* = 107) or placebo (*n* = 105). For the three coprimary endpoints and Epworth Sleepiness Scale, all three doses of ON-SXB demonstrated clinically meaningful, statistically significant improvement versus placebo (all *p* < 0.001). For ON-SXB 9 g versus placebo, increase in mean sleep latency was 10.8 versus 4.7 min (Least squares mean difference, LSMD [95% CI], 6.13 [3.52 to 8.75]), 72.0% versus 31.6% were rated much/very much improved on Clinical Global Impression-Improvement (OR [95% CI], 5.56 [2.76 to 11.23]), change in mean weekly number of cataplexy attacks was –11.5 versus –4.9 (LSMD [95% CI], –6.65 [–9.32 to –3.98]), and change in Epworth Sleepiness Scale was –6.5 and –2.7 (LSMD [95% CI], –6.52 [–5.47 to –2.26]). Common adverse reactions included nausea, vomiting, headache, dizziness, and enuresis.

**Conclusions:**

ON-SXB significantly improved narcolepsy symptoms; its safety profile was consistent with SXB. ON-SXB conferred efficacy with a clearly beneficial single nighttime dose.

**Clinical Trial Registration:**

ClinicalTrials.gov: NCT02720744, https://clinicaltrials.gov/ct2/show/NCT02720744.

**Statement of Significance**

The results from the phase 3 REST-ON clinical trial demonstrated clinically meaningful improvements with FT218, a novel once-nightly formulation of sodium oxybate (SXB), in patients with narcolepsy and may represent a significant advancement in the standard of care for adults with narcolepsy compared to other SXB formulations by avoiding a second nightly dose.

## Introduction

Narcolepsy is a chronic disease characterized by the symptom pentad of excessive daytime sleepiness (EDS), cataplexy, disrupted nighttime sleep (DNS), sleep paralysis, and hypnagogic/hypnopompic hallucinations [1, 2]. Narcolepsy is a debilitating condition affecting approximately 1 in 2,000 people [3] that can have severe health and economic consequences for patients, with reduced quality of life [4–7]. People with narcolepsy may experience difficulties with employment, school attendance, caring for their families, social situations, and relationships [8]. Patients report that narcolepsy is also associated with the stigma of being considered lazy, careless, or incapable by others in their lives [8].

Narcolepsy is categorized into two subtypes: narcolepsy type 1 (NT1; previously termed narcolepsy with cataplexy) and type 2 (NT2; previously called narcolepsy without cataplexy) [9]. Both are characterized by severe EDS, short sleep latency, and sleep-onset rapid eye movement periods on the multiple sleep latency test [9]. However, they differ in pathophysiology: NT1 is characterized by low cerebrospinal fluid (CSF) hypocretin-1 levels, whereas NT2 is characterized by normal CSF hypocretin-1 levels [9].

For people with narcolepsy, symptoms are typically managed both behaviorally and pharmacologically [10–12]. Treatment regimens consist of stimulant/wake-promoting drugs (modafinil, armodafinil, solriamfetol, methylphenidate, amphetamines) to decrease EDS; antidepressants (mostly serotoninergic and noradrenergic) for cataplexy; pitolisant for EDS and cataplexy; and sodium oxybate (SXB) for EDS, cataplexy, and DNS [12]. SXB, the sodium salt of γ-hydroxybutyrate (GHB), is the only drug that has demonstrated efficacy across multiple narcolepsy symptoms, including DNS [10–14]. Owing to its sedating nature, SXB is taken at the bedside [10]. Calcium, magnesium, potassium, and sodium oxybates, or “mixed-salts” oxybate, was introduced in 2020 [15]. At present, the only approved formulations of oxybate in the United States and Europe require twice-nightly dosing, with a second dose taken 2.5 to 4 h after the first dose [15, 16], attributable to the short GHB half-life of 30 min to 1 h and the immediate-release formulation.

Patient adherence to medication regimens is recognized to be inversely correlated to dosing frequency, as has been demonstrated across multiple disease states including depression, schizophrenia, epilepsy, type 2 diabetes mellitus, cardiovascular disease, and chronic obstructive pulmonary disease, as well as with HIV treatment [17–24]. These and other therapeutic areas are replete with once-daily options, as opposed to twice-daily or thrice-daily formulations, often due to the application of modified-release technology. Although a medication that is taken twice daily (i.e. morning and evening) is more challenging than a once-daily medication, it would be even more problematic for a chronic medication requiring middle-of-the-night awakening. For patients with narcolepsy, overall medication adherence is suboptimal. One study reported that good adherence (≥80%) was observed for only 55.2% of patients, while 12.9% were intermediately adherent (51%–79%) and 31.9% were poorly adherent (≤50%) [25].

This forced awakening is disruptive to some patients and bed partners, especially considering that patients with narcolepsy typically already experience sleep fragmentation and poor sleep quality [8, 26]. In a surveillance study of 670 EU patients receiving twice-nightly SXB, 27.1% did not take SXB according to the recommended time schedule (i.e. 2.5 to 4 h after the first dose) [27]. This nonadherence to the prescribed dosing regimen was reported to occur daily for 7.2% of participants, a few days per week for 6.4% of participants, and a few days per month or less for 13.3% of participants [27]. Treatment-emergent adverse events (TEAEs) rated by the investigator as being due to this nonadherence were nausea, decreased appetite, cataplexy, dizziness, somnolence, abnormal behavior, and night sweats [27]. A serious AE (SAE) resulting in hospitalization was reported due to accidentally ingesting the second dose of SXB immediately after the first dose. Similarly, inadvertent double dosing, also resulting in hospitalization, was reported for one patient in a more recent randomized clinical trial of mixed-salts oxybate (*N* = 201) [28]. Although the labeling advises patients to remain in bed to take the second dose, falls leading to injury, and in some cases, hospitalization, have been reported when patients rise from bed [15]. Given these concerns, development of new formulations that minimize these negative effects is warranted.

Despite the twice-nightly dosing regimen, treatment with SXB has been transformational for patients living with narcolepsy who are able to comply with the regimen [8]. Because of inherent challenges with the drug moiety, the ability to formulate a modified-release delivery has, until recently, been elusive. However, a proprietary drug delivery technology has now been successfully applied to SXB, validated by extensive phase 1 pharmacokinetic testing [29]. Briefly, this technology provides an early single peak, following by a gradual decline in GHB concentration, corresponding to the negligible blood levels 8 h after the single bedtime dose. Theoretically, the pharmacokinetic profile may more closely approximate “normal” sleep, allowing for more slow-wave sleep (SWS) in the first half of the night, in contrast to twice-nightly SXB, in which the second segment of the night, associated with the second maximum concentration (*C*_max_), has more SWS [30]. FT218 is an investigational, extended-release, once-nightly formulation of SXB (ON-SXB) that uses this proprietary drug delivery technology. The premeasured dosing packets contain a mix of immediate-release and controlled-release microparticles of SXB. One 6-g dose of ON-SXB has been shown to be equivalent in systemic drug exposure to immediate-release SXB given as two separate 3-g doses [29]. This phase 3 trial evaluated the efficacy and safety of ON-SXB in patients with NT1 and NT2.

## Methods

### Study design

**Figure 1.**
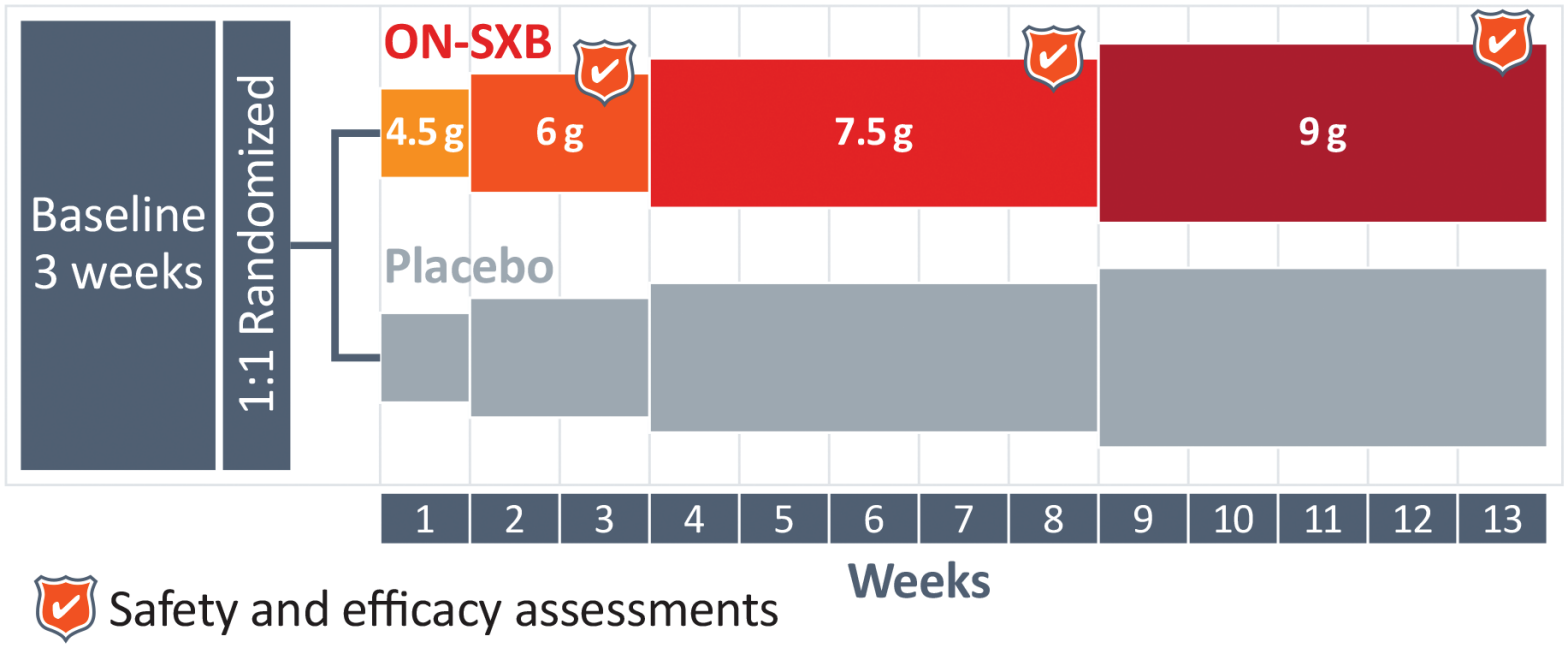
Study design.REST-ON is a 13-week, phase 3, double-blind, multicenter trial with a 1:1 randomization to ON-SXB vs. placebo. Participants receiving ON-SXB underwent a forced titration from 4.5 g (week 1) to 6 g (weeks 2–3) to 7.5 g (weeks 4–8), and finally 9 g (weeks 9–13). Randomization was stratified according to narcolepsy type (NT1/NT2). There was a 3-week baseline screening period before randomization. MWT, number of cataplexy attacks, ESS, and PSG were assessed at baseline and at weeks 3, 8, and 13 of treatment. Number of cataplexy attacks, hypnagogic hallucinations, and sleep paralysis events were recorded in the daily sleep and symptom diaries, which were reviewed at weeks 3, 8, and 13. CGI-S (for sleepiness) was recorded at baseline; CGI-I was recorded at weeks 3, 8, and 13. Adverse events were documented at weeks 3, 8, and 13, but could be reported at any time during the trial. CGI-I, Clinical Global Impression of Improvement; CGI-S, Clinical Global Impression of Severity; ESS, Epworth Sleepiness Scale; MWT, Maintenance of Wakefulness Test; NT1, narcolepsy type 1; NT2, narcolepsy type 2; ON-SXB, once-nightly sodium oxybate; PSG, polysomnography.

REST-ON was a phase 3, double-blind, placebo-controlled, 2-arm, multicenter, randomized clinical trial (NCT02720744) designed to investigate the efficacy and safety of ON-SXB for the treatment of EDS and cataplexy in patients with narcolepsy. The trial comprised a 3-week screening period, a 13-week treatment period, and a 1-week follow-up period (Figure 1). Participants were randomly assigned 1:1 to ON-SXB or placebo; randomization was stratified by narcolepsy type (NT1 or NT2). Participants initially received 4.5 g for 1 week, followed by 6 g for weeks 2 to 3, 7.5 g for weeks 4 to 8, and then 9 g for weeks 9 to 13. This study design allowed evaluation of efficacy and safety of each dose, within the same participants, with a titration scheme similar to that of the approved twice-nightly product.

REST-ON was conducted in adherence to the ethical principles of the Declaration of Helsinki, Good Clinical Practice guidelines, International Council for Harmonisation guidelines, and any applicable national and local laws and regulatory requirements. The protocol was approved by the centers’ investigational review boards. All participants provided written informed consent before participation; for participants 16 or 17 years of age, consent was obtained from both the participant and their legally authorized representative.

### Participants

Participants ≥16 years of age with documented evidence of a diagnosis of NT1 or NT2 as defined by the International Classification of Sleep Disorders-3 criteria [31] were eligible. Additional key inclusion criteria were current continuing presence of EDS as defined by patient report for the past 3 months, Epworth Sleepiness Scale (ESS) > 10, and current continuing presence of cataplexy as defined by patient report for the last 3 months (NT1 only). Concomitant stimulants were permitted if participants were on a stable dosing regimen for at least 3 weeks before screening and throughout the entire study period; anticataplexy drugs were not permitted, and participants who were on these medications were tapered off in a 3-week washout before the screening period. Female participants of childbearing potential must have agreed to practice effective methods of contraception. For randomization, participants were required to have baseline Maintenance of Wakefulness Test (MWT) mean sleep latency < 11 min following baseline polysomnography (PSG). Those with NT1 were required to demonstrate current continuing presence of cataplexy, as defined by an average of 8 reported cataplexy attacks per week in the screening/baseline sleep and symptom diary.

Concurrent use of the following was prohibited: experimental medications (e.g. pitolisant when REST-ON was conducted), sodium valproate, anticonvulsants, clonidine, selective serotonin reuptake inhibitors, serotonin-norepinephrine reuptake inhibitors, monoamine oxidase inhibitors, tricyclic antidepressants, hypnotics, anxiolytics, sedating antihistamines, and antipsychotics. Diagnosis of moderate and severe sleep apnea or other sleep disorder known to cause EDS, as determined by PSG and sleep history, including any PSG results indicating an apnea-hypopnea index (AHI) ≥ 15, or AHI < 15/h requiring continuous positive airway pressure, was exclusionary. Overnight PSG was performed in the clinic (not home study) at baseline to exclude AHI > 15. A hypopneic event was considered to occur when there was a peak signal excursion drop by >30% of pre-event baseline using nasal pressure or an acceptable alternative signal (e.g. respiratory inductance plethysmography sum) lasting >10 s. The event must have had a >3% oxygen desaturation or the event was associated with an arousal. Additional key exclusion criteria were presence of any unstable or clinically significant medical and psychiatric disorders that may put the patient at risk; previous history or current suicidal ideation; history of drug or alcohol abuse; pregnancy or breastfeeding; smoking during the night; history of seizure disorder, head trauma, or past invasive intracranial surgery; severe chronic obstructive pulmonary disease; other underlying respiratory and/or other underlying condition or disorder that would potentiate risk of respiratory or central nervous system depression with concomitant use of SXB; and uncontrolled hypertension. Initially, prior use of SXB was exclusionary; the protocol was amended in 2018 to allow prior use of SXB ≤ 4.5 g per night for <2 weeks and ≥1 year before study entry (only one participant was enrolled who met these criteria). At the time that REST-ON was conducted, mixed-salts oxybate was investigational.

### Assessments

The primary objective of the trial was to assess the efficacy of 9, 7.5, and 6 g of ON-SXB versus placebo in treating EDS in participants with NT1 or NT2 and cataplexy in participants with NT1. The coprimary endpoints were change from baseline in mean sleep latency on the MWT, in investigator-assessed rating of “much improved” or “very much improved” on the Clinical Global Impression of Improvement (CGI-I), and in the mean weekly number of cataplexy attacks. Safety and efficacy assessments were taken at baseline and at weeks 3, 8, and 13. The MWT measured the mean sleep latency across five trials that were terminated after 30 min if no sleep onset occurred or immediately after sleep onset, defined as the first epoch of any stage of sleep, averaged over the test day. The 30-minute duration achieves the appropriate balance between the 20-minute and 40-minute intervals used in prior studies. PSG was performed the night before each MWT. CGI-I was the clinician’s global impression of improvement in overall condition compared to baseline. CGI-I was evaluated on a 7-point Likert scale ranging from 1 (“very much improved”) to 7 (“very much worse”). For participants with NT1, the number of cataplexy attacks per week was recorded daily in the sleep and symptom daily diary, with attacks recorded as 0, 1, 2, 3, 4, or ≥5 per day. The sleep diary was recorded on a mobile phone with a preloaded app. A minimum of three entries per week was required for the average to be considered an observation.

Secondary endpoints included other measures of efficacy (DNS, ESS, nocturnal arousals, sleep paralysis, hypnagogic hallucination, and sleep quality) of 9, 7.5, and 6 g of ON-SXB versus placebo in patients with NT1 and NT2, and safety. The ESS is a questionnaire that evaluates extent of subjective sleepiness in everyday situations [32]. Patients were asked to rate their likelihood of dozing during eight activities on a scale from 0 (“never”) to 3 (“high”). All other secondary endpoints, as well as post hoc analyses of efficacy and safety in the NT1 and NT2 cohorts, will be reported elsewhere.

### Statistical analysis

Participants were enrolled and randomly assigned to the ON-SXB or placebo arm to achieve the number of completers needed to reach 140 (70 per arm) for MWT/CGI-I and 100 (50 per arm) for cataplexy to reach a two-sided alpha of 0.05, which yielded an overall 81% power for all three endpoints. Efficacy was evaluated in the modified intent-to-treat population (mITT), defined as all randomized participants with ≥1 efficacy measurement after receiving the 6-g dose (ON-SXB or placebo). A mixed-effects model for repeated measures (MMRM) was used to analyze change from baseline for the MWT and cataplexy endpoints. Each model included treatment, time (visit at which the measurement was taken), treatment-by-time interaction, site (United States or non-United States) and baseline score as fixed effects, and participants as random effects. For CGI-I, a GLIMMIX model for binomial data with logit link was used to analyze the categorized CGI-I response, that is, the proportion of participants who were very much or much improved. The observed values for the categorized response were used as responses in the model. Similar to the MMRM, the GLIMMIX model included treatment, time (at which the measurement was taken), treatment-by-time interaction and site (United States or non-United States) as fixed effects and participants as random effects. In fitting the model, time ranged over the entire period of observations. Permutation tests were performed based on the primary MMRM model, and statistical significance was determined by the tails of the distribution of *p* values. Least squares mean differences (LSMDs), odds ratios, associated 95% CIs, and *p* values were calculated. All statistical tests were performed using the two-sided alpha level of 0.05 unless otherwise specified. Testing was performed in a hierarchical manner for MWT, then the CGI-I, and then cataplexy, in that order, at 9, 7.5, and 6 g. If the 9-g dose demonstrated efficacy (*p* < 0.05) for all three endpoints, the same sequence of testing was followed for the 7.5-g dose. If the 7.5-g dose demonstrated efficacy (*p* < 0.05) for all three endpoints, the same sequence of testing was followed for the 6-g dose.

All participants who received ≥1 dose of study drug were included in the safety population. Adverse event data were obtained at all study visits from the time of informed consent until 7 days after the last dose of study drug was taken. TEAEs were defined as those occurring or increasing in severity after the first dose of study drug was taken. Each TEAE was assessed as mild, moderate, or severe in intensity. The relationship of TEAEs to study drug was assessed by the investigators using the following categories: not related, unlikely related, possibly related, and related. They were summarized based on number and percentages of participants with any TEAEs, SAEs, adverse drug reactions (ADRs; defined as adverse events assessed by the investigator to be related or possibly related to study drug), and permanent withdrawal from the study. Vital signs were taken at each study visit; clinical laboratory values were assessed at baseline and end of study.

## Results

### Patient disposition and demographics

**Figure 2.**
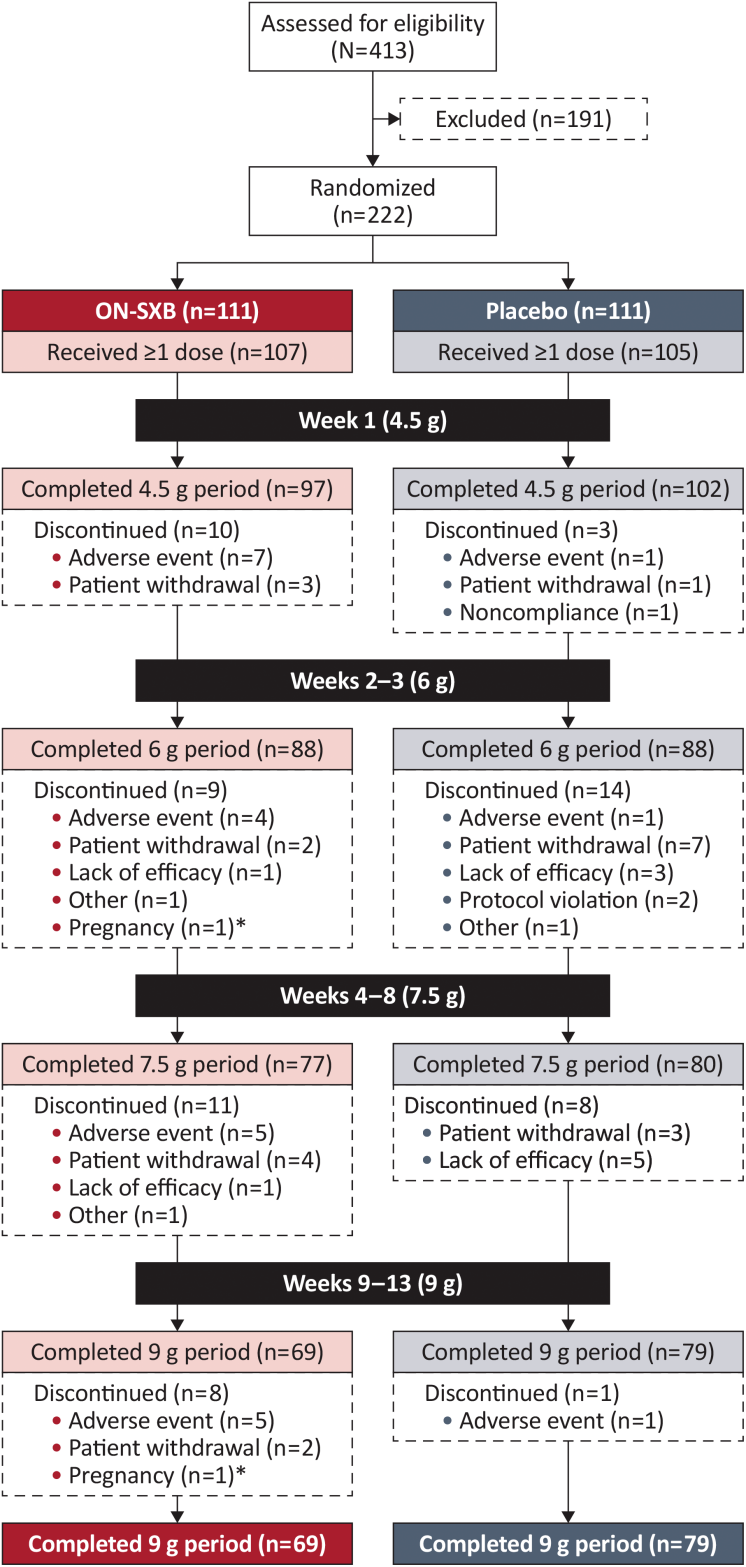
Patient disposition.CONSORT diagram of patient disposition. ON-SXB, once-nightly sodium oxybate. *Terminated.

REST-ON was conducted from November 17, 2016, to March 25, 2020, at 71 centers in Australia, Canada, Czech Republic, Denmark, Finland, France, Germany, Netherlands, Switzerland, United Kingdom, and United States. A total of 413 participants were assessed for eligibility. Of these, 191 were excluded for not meeting one or more screening or randomization criteria; common reasons for exclusion included not meeting randomization EDS confirmation criteria (*n* = 59), unwillingness to comply with study mandates and procedures (*n* = 27), sleep apnea (*n* = 24), history of drug or alcohol abuse (*n* = 17), not meeting cataplexy criteria at randomization (*n* = 15) or screening (*n* = 14), and not having documented evidence of NT1 or NT2 (*n* = 14). In total, 222 were randomized 1:1 to the ON-SXB arm or the placebo arm (*n* = 111 each; Figure 2); 212 received at least one dose of study drug. Sixty-four (30.2%) participants discontinued the trial (ON-SXB, *n* = 38 [35.5%]; placebo, *n* = 26 [24.8%]). The most common reasons for discontinuation were TEAEs (overall, *n* = 24 [11.3%]; ON-SXB, *n* = 21 [19.6%]; placebo, *n* = 3 [2.9%]) and participant withdrawal (overall, *n* = 22 [10.4%]; ON-SXB, *n* = 11 [10.3%]; placebo, *n* = 11 [10.5%]). In total, 148 (69.8%) participants completed the study (ON-SXB, *n* = 69 [64.5%]; placebo, *n* = 79 [75.2%]).

Baseline demographics were well balanced between the ON-SXB and placebo arms. The mean (SD) age was 31 (11) and 32 (11) years in the ON-SXB and placebo arms, respectively (Table 1). Most participants were female (ON-SXB, 64.5%; placebo, 71.4%) and white (ON-SXB, 74.8%; placebo, 76.2%). Approximately 75% of participants had NT1 (ON-SXB, 74.8%; placebo, 78.1%). During the trial, 171 (80.7%) participants were taking ≥1 concomitant medication; the most frequently reported concomitant medications were stimulants. In the mITT population, 66 participants in the ON-SXB arm and 53 in the placebo arm were taking concomitant stimulants (modafinil [ON-SXB, 21.5%; placebo, 21.0%], armodafinil [ON-SXB, 12.1%; placebo, 6.7%], amphetamine [various; ON-SXB, 10.3%; placebo, 5.7%], and methylphenidate [ON-SXB, 10.3%; placebo, 6.7%]). Other frequently reported concomitant medications were over-the-counter analgesics (ibuprofen, acetaminophen) and vitamins. Hypertension was not an exclusion criterion; approximately 8% of participants enrolled had a medical history of hypertension. Only one patient had received prior SXB treatment.

**Table 1.** Baseline demographics and disease characteristics (safety population)

Characteristic	ON-SXB *n* = 107	Placebo *n* = 105
Mean age (range), years	30.9 (16–72)	31.6 (16–69)
Sex, *n* (%)		
Female	69 (64.5)	75 (71.4)
Male	38 (35.5)	30 (28.6)
Race, *n* (%)		
White	80 (74.8)	80 (76.2)
Black/African American	21 (19.6)	15 (14.3)
Asian	3 (2.8)	8 (7.6)
Other*	3 (2.8)	2 (1.9)
Region, *n* (%)		
United States	63 (58.9)	53 (50.5)
Rest of world	44 (41.1)	52 (49.5)
Median BMI (range), kg/m^2^	26.1 (16.9–71.9)	26.4 (18.1–46.5)
Mean BMI (SD), kg/m^2^	28.1 (7.8)	28.2 (6.6)
Narcolepsy type, *n* (%)		
NT1	80 (74.8)	82 (78.1)
NT2	27 (25.2)	23 (21.9)
Hypertension, *n* (%)	10 (9.3)	6 (5.7)

*Egyptian (*n* = 2), white, American Indian/Alaska Native (*n* = 1), half Asian, half white (*n* = 1), and multiracial (white/African American/Native American, *n* = 1).

BMI, body mass index; NT1, narcolepsy type 1; NT2, narcolepsy type 2; ON-SXB, once-nightly sodium oxybate.

## Efficacy

### Coprimary endpoints

#### Maintenance of Wakefulness Test.

**Figure 3.**
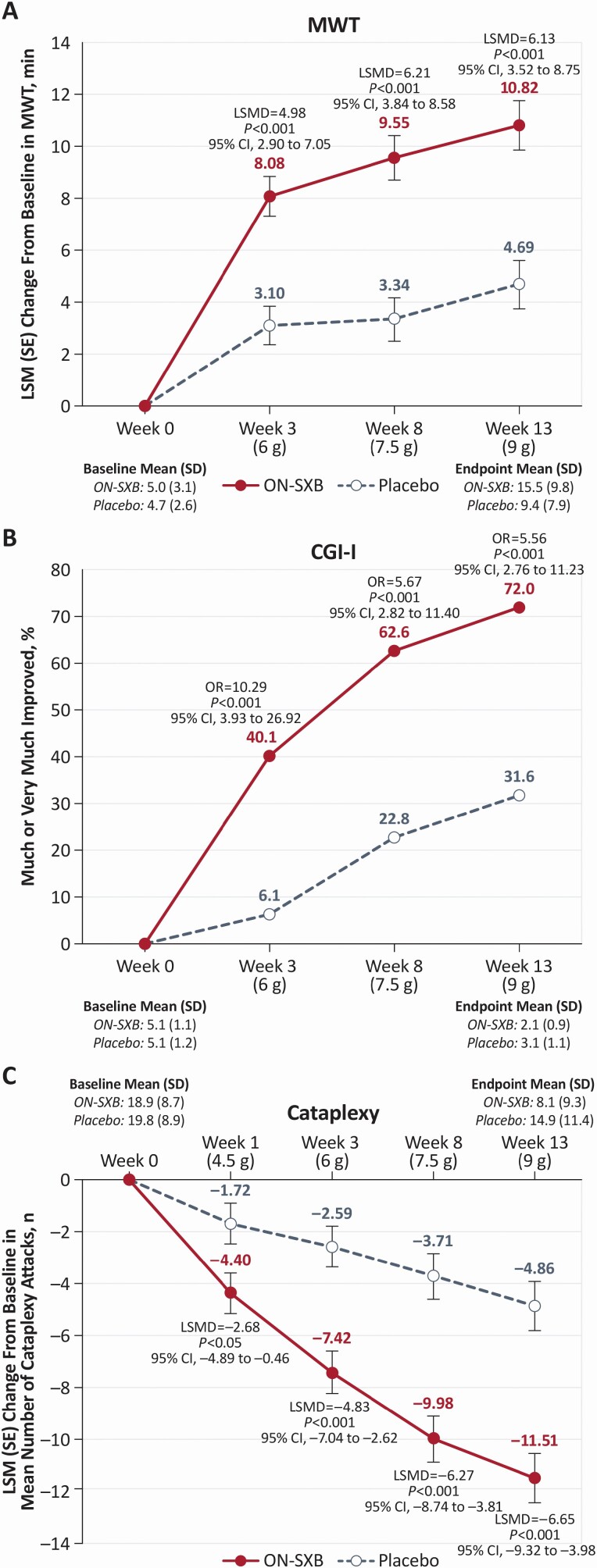
Change from baseline for the three coprimary endpoints (mITT population).A mixed-effects model for repeated measures was used for MWT and cataplexy analyses. A GLIMMEX model was used for CGI-I analysis. (A) LSM change from baseline in the MWT for patients receiving ON-SXB or matching placebo. (B) Percentage of patients rated much or very much improved on the CGI-I for patients receiving ON-SXB or matching placebo. (C) LSM change from baseline in mean number of cataplexy attacks for patients receiving ON-SXB or matching placebo. CGI-I, Clinical Global Impression of Improvement; LSM, least squares mean; LSMD, least squares mean difference; mITT, modified intent to treat; MWT, Maintenance of Wakefulness Test; ON-SXB, once-nightly sodium oxybate; OR, odds ratio.

Mean sleep latency on the MWT was similar between the ON-SXB and placebo arms at baseline (5.0 and 4.7 min, respectively). The increase in sleep latency was significantly greater with ON-SXB vs placebo at week 3 for the 6-g dose (least squares mean change from baseline, 8.1 vs. 3.1 min, respectively; LSMD [95% CI], 4.98 [2.90 to 7.05]; *P*<0.001); at week 8 for the 7.5-g dose (9.6 vs. 3.3 min, respectively; LSMD [95% CI], 6.21 [3.84 to 8.58]; *P*<0.001); and at week 13 for the 9-g dose (10.8 vs. 4.7 min, respectively; LSMD [95% CI], 6.13 [3.52 to 8.75]; *p* < 0.001) (all Figure 3A).

#### Clinical Global Impression-Improvement.

The mean baseline CGI-Severity was 5.1 in both treatment arms, indicating that participants were markedly impaired. A significantly greater proportion of participants in the ON-SXB treatment arm versus placebo were rated much or very much improved on the CGI-I at week 3 for the 6-g dose (40.1% vs. 6.1%, respectively; odds ratio [95% CI], 10.29 [3.93 to 26.92]; *p* < 0.001); at week 8 for the 7.5-g dose (62.6% vs. 22.8%, respectively; odds ratio [95% CI], 5.67 [2.82 to 11.40]; *p* < 0.001); and at week 13 for the 9-g dose (72.0% vs. 31.6%, respectively; odds ratio [95% CI], 5.56 [2.76 to 11.23]; *p* < 0.001) (all Figure 3B).

#### Number of cataplexy attacks.

The mean baseline number of weekly cataplexy attacks was similar in the ON-SXB and placebo arms (18.9 and 19.8, respectively). The decrease in least squares mean change from baseline in the number of weekly cataplexy attacks was significantly greater with ON-SXB vs placebo at week 3 for the 6-g dose (–7.4 vs. –2.6, respectively; LSMD [95% CI], –4.83 [–7.04 to –2.62]; *p* < 0.001); at week 8 for the 7.5-g dose (–10.0 vs. –3.7, respectively; LSMD [95% CI], –6.27 [–8.74 to –3.81]; *p* < 0.001); and at week 13 for the 9-g dose (–11.5 vs. –4.9, respectively; LSMD [95% CI], –6.65 [–9.32 to – 3.98]; *p* < 0.001) (all Figure 3C). Although not a prespecified endpoint, a post hoc analysis revealed that significantly greater least squares mean change from baseline was also observed at week 1 with 4.5 g ON-SXB vs placebo (least squares mean change, –4.4 vs. –1.7; LSMD [95% CI], –2.68 [–4.89 to –0.46]; *p* < 0.05; Figure 3C).

### Secondary endpoint

#### Epworth Sleepiness Scale.

**Figure 4.**
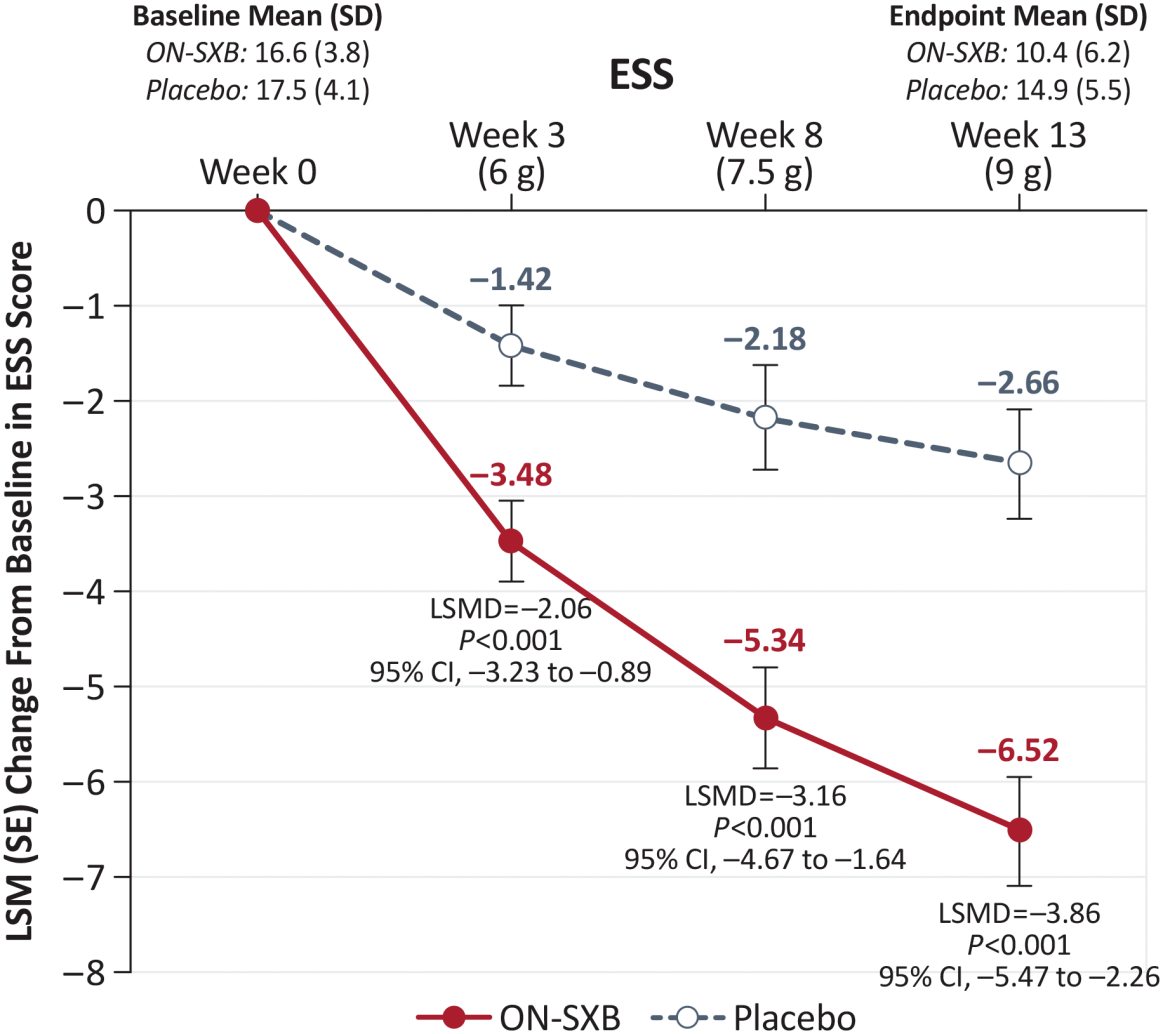
Change from baseline in ESS (mITT population).A mixed-effects model for repeated measures was used for the analysis. LSM change from baseline in the ESS for patients receiving ON-SXB or matching placebo. ESS, Epworth Sleepiness Scale; LSM, least squares mean; LSMD, least squares mean difference; mITT, modified intent to treat; ON-SXB, once-nightly sodium oxybate.

At baseline, ESS score was similar in the ON-SXB and placebo arms (16.6 and 17.5, respectively). The decrease in least squares mean change from baseline was significantly greater with ON-SXB versus placebo at week 3 for the 6-g dose (–3.5 vs. –1.4, respectively; LSMD [95% CI], –2.06 [–3.23 to –0.89]; *p* < 0.001); at week 8 for the 7.5-g dose (–5.3 vs. –2.2, respectively; LSMD [95% CI], –3.16 [–4.67 to –1.64]; *p* < 0.001); and at week 13 for the 9-g dose (–6.5 vs. –2.7, respectively; LSMD [95% CI], –3.86 [–5.47 to –2.26]; *p* < 0.001) (all Figure 4).

### Safety

**Figure 5.**
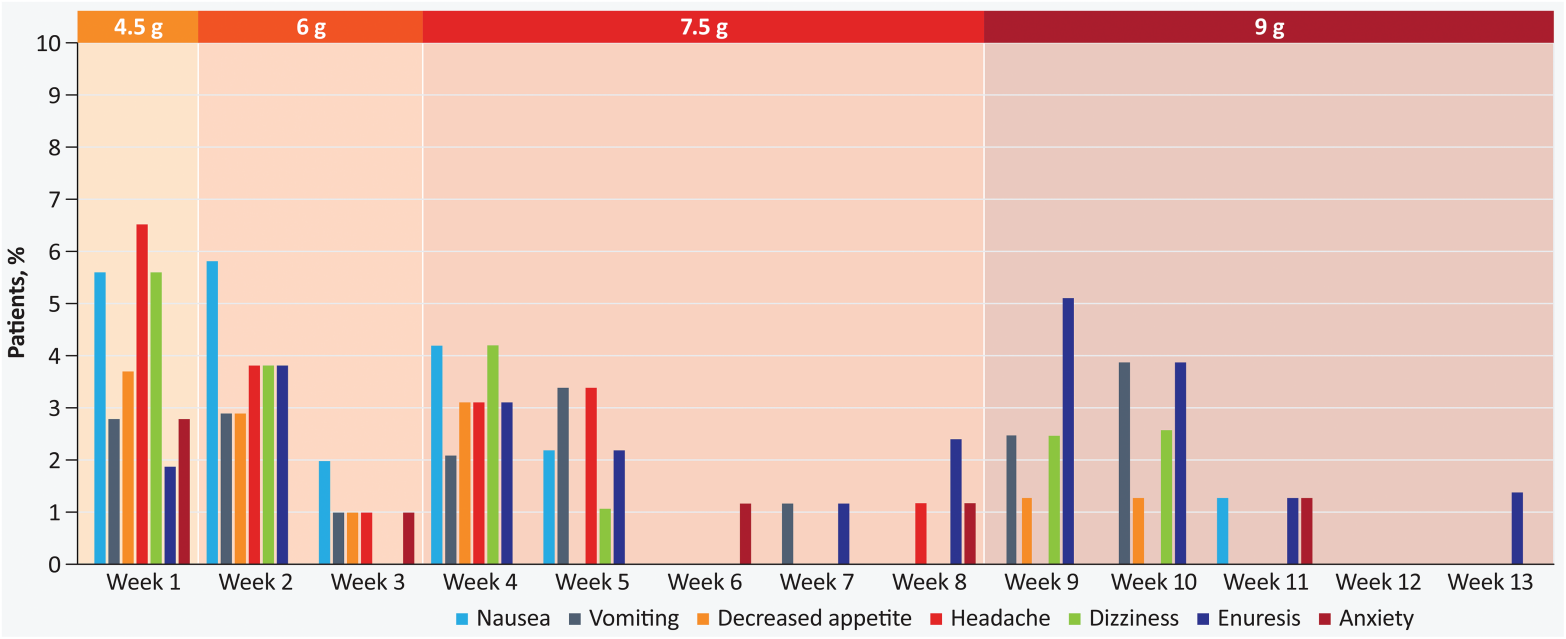
Incidence of related TEAEs over time for ON-SXB.ON-SXB, once-nightly sodium oxybate; TEAE, treatment-emergent adverse event.

In the safety population, 83 (77.6%) and 49 (46.7%) participants in the ON-SXB and placebo arms, respectively, experienced a TEAE (Table 2). Of these, most were considered mild (ON-SXB, *n* = 32 [29.9]; placebo, *n* = 24 [22.9]) or moderate (ON-SXB, *n* = 40 [37.4]; placebo, *n* = 20 [19.0]) in severity. The most frequently reported TEAEs for all participants treated with ON-SXB, at rates higher than placebo, were nausea (22.4%), headache (18.7%), vomiting (17.8%), dizziness (15.9%), enuresis (15.9%), decreased appetite (12.1%), anxiety (7.5%), hyperhidrosis (5.6%), and decreased weight (5.6%). In the ON-SXB arm, when looking across the three dosing periods, rates of known SXB AEs (i.e. nausea, vomiting, somnolence, dizziness, enuresis) were low (Table 2) and there was no dose-response relationship, with the exception of enuresis (Figure 5). No participants in the ON-SXB arm and one participant in the placebo arm discontinued due to a TEAE of worsening sleep apnea. A post hoc analysis of PSG data showed a similar decrease from baseline in the AHI at all doses in both the ON-SXB and placebo arms.

**Table 2.** Safety summary (safety population)

	Week 1		Weeks 2–3		Weeks 4–8		Weeks 9–13	
*n* (%)	ON-SXB 4.5 g (*n* = 107)	Placebo (*n* = 105)	ON-SXB 6 g (*n* = 97)	Placebo (*n* = 102)	ON-SXB 7.5 g (*n* = 88)	Placebo (*n* = 88)	ON-SXB 9 g (*n* = 77)	Placebo (*n* = 80)
Any TEAE	37 (34.6)	14 (13.3)	38 (39.2)	21 (20.6)	42 (47.7)	24 (27.3)	43 (55.8)	20 (25.0)
Any serious AE	1 (0.9)	1 (1.0)	1 (1.0)	0	1 (1.0)	0	2 (2.6)	1 (1.3)
TEAE leading to discontinuation	7 (6.5)	1 (1.0)	6 (6.2)	1 (1.0)	5 (5.7)	1 (1.1)	5 (6.5)	0
Any ADR*	30 (28.0)	9 (8.6)	28 (28.9)	4 (3.9)	30 (34.1)	6 (6.8)	27 (35.1)	4 (5.0)
ADR leading to discontinuation	6 (5.6)	0	4 (4.1)	1 (1.0)	4 (4.5)	1(1.1)	3 (3.9)	0
Common ADRs†								
Vomiting	3 (2.8)	1 (1.0)	3 (3.1)	1 (1.0)	5 (5.7)	0	4 (5.2)	0
Nausea	6 (5.6)	1 (1.0)	8 (8.2)	2 (2.0)	6 (6.8)	0	1 (1.3)	1 (1.3)
Weight decreased	1 (0.9)	0	0	0	0	0	3 (3.9)	0
Decreased appetite	4 (3.7)	0	4 (4.1)	0	3 (3.4)	0	2 (2.6)	0
Dizziness	6 (5.6)	0	4 (4.1)	0	5 (5.7)	0	4 (5.2)	0
Somnolence	0	1 (1.0)	1 (1.0)	0	2 (2.3)	0	3 (3.9)	0
Headache	7 (6.5)	4 (3.8)	5 (5.2)	1 (1.0)	5 (5.7)	1 (1.1)	0	1 (1.3)
Enuresis	2 (1.9)	0	4 (4.1)	0	8 (9.1)	0	7 (9.1)	0
Anxiety	3 (2.8)	0	0	0	3 (3.4)	1 (1.1)	1 (1.3)	0
Somnambulism	1 (0.9)	0	2 (2.1)	0	0	0	0	0

*ADRs defined as adverse events assessed by the investigator to be related or possibly related to study drug.

^†^Occurring in ≥2% of patients receiving ON-SXB and more frequently in the ON-SXB group vs. placebo.

ADR, adverse drug reaction; AE, adverse event; ON-SXB, once-nightly sodium oxybate; TEAE, treatment-emergent adverse event.

Serious TEAEs were reported by seven participants (3.3%) overall (ON-SXB arm, *n* = 5 [4.7%]; placebo arm, *n* = 2 [1.9%]). The five SAEs in the ON-SXB arm occurred across dose groups (1 each in the 4.5-g [diabetes mellitus inadequate control], 6-g [paresthesia], 7.5-g [perirectal abscess], and 9-g [suicidal ideation] dose groups; 1 in the posttreatment follow-up period [hypertension]). Only the SAE in the 9-g dose group (suicidal ideation) was considered treatment-related. This participant had a history of depression and anxiety; study drug was discontinued and the participant spent two nights in a psychiatric ward, where she was treated with lorazepam and then discharged with a lorazepam prescription. One week after reporting suicidal ideation, she was evaluated by the investigator. Her Columbia Suicide Severity Rating Scale score was negative; as such, the SAE was considered resolved. No deaths were reported in the study.

Sixty-one (57.0%) participants in the ON-SXB arm and 17 (16.2%) participants in the placebo arm experienced ADRs. Overall, 17 (15.9%) participants in the ON-SXB arm and 2 (1.9%) participants in the placebo arm discontinued owing to ADRs. ADRs leading to discontinuation in ON-SXB–treated participants were dizziness (*n* = 5 [4.7%]); nausea (*n* = 3 [2.8%]); anxiety (*n* = 3 [2.8%]); headache (*n* = 2 [1.9%]); and vomiting, diarrhea, dysphagia, somnolence, abnormal dreams, delirium, depressed mood, depression, enuresis, restlessness, suicidal ideation, dyspnea, and painful respiration (*n* = 1 each [0.9%]). The most commonly reported ADRs (>5%) in the ON-SXB treatment arm at the 9-g dose were enuresis (9.1%), dizziness (5.2%), and vomiting (5.2%) (Table 2).

There were no clinically meaningful changes from baseline in clinical laboratory values, blood pressure, or heart rate for ON-SXB or clinically meaningful differences compared to placebo. Mean change from baseline in weight at week 13 was ‒1.29 kg and 0.19 kg in the ON-SXB and placebo arms, respectively. In a post hoc analysis, 17.5% of patients receiving ON-SXB versus 3.8% of patients receiving placebo had ≥5% weight loss at week 13. Least squares mean (SE) change from baseline to week 13 in body mass index was ‒0.51 (0.13) kg/m^2^ for patients receiving ON-SXB and 0.08 (0.13) kg/m^2^ for patients receiving placebo (LSMD [95% CI], ‒0.59 [‒0.95 to ‒0.23]; *p* = 0.001).

## Discussion

ON-SXB demonstrated statistically significant (*p* < 0.001) and clinically meaningful improvement for the three prespecified coprimary endpoints compared to placebo at all evaluated doses (6 g, 7.5 g, and 9 g): objective EDS assessed by MWT, frequency of cataplexy, and overall clinical condition as measured by the CGI-I. Statistically significant (*p* < 0.001) and clinically meaningful improvement was also demonstrated for the secondary endpoint of subjective EDS assessed by ESS in patients with narcolepsy with or without cataplexy. In contrast, in the 2002 twice-nightly SXB pivotal clinical trial, only the 9-g dose was statistically significant on EDS as measured by the ESS [33]. Newly released clinical practice guidelines from the American Academy of Sleep Medicine set a change from baseline compared to placebo of two or more minutes on the MWT, including the 95% CIs, as being the clinical significance threshold [34, 35]; ON-SXB achieved this standard for all three doses evaluated (Figure 3A). This is the first randomized controlled trial that demonstrated the efficacy and safety of a 7.5-g dose of SXB; given that the efficacy was nearly comparable to the 9-g dose compared to placebo, this may afford confidence for clinicians and patients that a lower dose can be effectively used in practice. Moreover, this is the first known publication describing a statistically significant reduction of cataplexy compared to placebo at week 1 with a 4.5-g dose of SXB. Supporting these prespecified findings in the overall narcolepsy cohort, post hoc analyses demonstrated consistent results for ON-SXB on EDS and overall condition, as assessed by MWT, ESS, and CGI-I, in both NT1 and NT2 subgroups and in subgroups of patients who were or were not receiving concomitant stimulant treatment [36, 37]; these analyses will be reported in future publications.

ON-SXB treatment (all doses, including at 3 weeks, with 6 g) was associated with significant improvement versus placebo on the ESS. In a recent study, change from baseline in the ESS score was shown to be significantly correlated with change in different measures of health-related quality of life, including the Functional Outcomes of Sleep Questionnaire, the Work Productivity and Activity Impairment questionnaire, and subscales of the Medical Outcomes Study 36-Item Short Form Health Survey, in patients with narcolepsy [5]. Thus, the improvement on the ESS scale observed with ON-SXB suggests that treatment with ON-SXB may correspond to improved quality of life for patients with narcolepsy; however, a direct measure of quality of life was not available for this study.

ON-SXB was generally well tolerated, with ADRs mostly rated mild or moderate in severity. Incidence of ADRs was higher early in the trial, increased with each dose increase, and then decreased over time, reflecting the attenuation of ADRs as participants adjusted to the medication (Table 2 and Figure 5). ADRs were consistent with known side effects of SXB [16, 33], and no new safety signals were observed. Enuresis was the only ADR with an apparent dose-response relationship, with a rate of 9.1% at the 9-g dose. Enuresis decreased as the time period and 9-g dosing period progressed. Although cross-trial comparisons should be interpreted with caution owing to different sample sizes and trial designs, labeling for the immediate-release oxybates reports a 3%–7% rate of enuresis [15, 16], and urine incontinence, which was reported as enuresis in most instances, occurred at a rate of 14.3% with the 9-g dose and 6.1% with the 6-g dose [33]. Other events that may result in discontinuation with conventional SXB are relevant. Among the known TEAEs with SXB, rates of headache, dizziness, and nausea appear to be lower with ON-SXB than with twice-nightly SXB across doses (headache: ON-SXB, 4%–8% and twice-nightly SXB, 9%–31%; dizziness: ON-SXB, 4%–6% and twice-nightly SXB, 7%–34%; nausea: ON-SXB, 4%–9% and twice-nightly SXB, 6%–34%) [33, 38].

Potential for lower rates of ADRs with ON-SXB compared to twice-nightly SXB may be due to several factors, including the lower overall *C*_max_, having only 1 *C*_max_ with ON-SXB versus 2 with twice-nightly SXB [29], and the slower titration scheme in the REST-ON design. In the ON-SXB phase 1 studies, the known SXB ADRs mostly occurred around *C*_max_ [39]. Therefore, having a single *C*_max_ instead of 2, as with twice-nightly SXB, may lead to lower rates of ADRs [39]. As head-to-head studies are not required for regulatory approval in the United States, this pivotal study was placebo-controlled; there are no planned head-to-head studies with either formulation of immediate-release, twice-nightly oxybate. Clinical practice will likely elucidate patient preference between a single bedtime dose and a chronic regimen requiring middle-of-the-night awakening for a second dose.

There were no clinically meaningful changes from baseline in hematology or clinical laboratory values, blood pressure, or heart rate with ON-SXB treatment. Weight loss, as evidenced by change from baseline in body mass index, was greater for participants treated with ON-SXB versus placebo; thus, for those who are overweight, ON-SXB may have a beneficial metabolic effect. These findings provide further support of the safety and tolerability of ON-SXB in patients with narcolepsy.

This trial had some limitations. The design of the sleep diary limited the number of cataplexy events reported to “5 or more per day,” which likely underreported the number of cataplexy attacks experienced by some participants and thus may have underestimated the effect of ON-SXB on this symptom. An increasing placebo effect was observed during the trial, which may have been due to investigator or patient expectation of benefit due to the increasing dose titration and a previously demonstrated dose-response relationship for efficacy with twice-nightly SXB. Although statistically significant improvement (*p* < 0.001 across all endpoints) was seen, this placebo effect may have resulted in underestimation of the effect of ON-SXB on the symptoms of narcolepsy. Approximately 70% of participants completed the 13-week trial. Dizziness, nausea, anxiety, and headache, which are well-known side effects of SXB, infrequently led to discontinuation. Sensitivity analyses using various methods of handling missing data affirmed the positive findings for all three doses and are planned to be further described in future publications. Of note, for the mixed-salts oxybate pivotal trial, the withdrawal design enrolled nearly 40% of participants who were already receiving, and therefore presumably tolerating, SXB [28]. Nevertheless, in the open-label titration phase and stable-dosing period, the discontinuation rate was 32% [28].

Modified-release formulations are commonly applied to therapeutics across disease states to decrease patient burden, improve adherence with a prescribed regimen, and ultimately enhance the overall patient experience [40, 41]. This is particularly important for chronic medical conditions such as narcolepsy, which is debilitating to every facet of a patient’s life when not successfully treated. ON-SXB has received orphan drug designation from the US Food and Drug Administration (FDA) based on the plausible hypothesis that it may be clinically superior to the twice-nightly formulation of SXB already approved for the same indication. Once-nightly dosing may result in better medication adherence for patients and may have a beneficial effect on nocturnal sleep. Additionally, ON-SXB will avoid the patient potentially awakening a bed partner to self-administer the second dose of twice-nightly SXB. The plasma concentrations decline steadily after the first, and only, *C*_max_, which occurs early in the night postdose, so patients can usually awaken when required for urination or other reasons. However, the reduced tendency to awaken at night may have some considerations. Patients should be advised to consider limiting fluid intake several hours before dosing, to void before dosing, and to stay in bed after dosing. In REST-ON, the protocol did not include these directions. Enuresis was reported at a rate of 9% in the 9-g dose; however, enuresis was largely limited to the first 2 weeks of the higher dose (Figure 5). Somnambulism occurred at a rate of 0.9% in the 4.5-g dose and 2.1% in the 6-g dose and was not reported with either the 7.5- or 9-g dose. In REST-ON, patients were instructed to stay in bed after dosing. However, two patients receiving ON-SXB and one receiving placebo experienced AEs of falls, which were nonserious in nature; it is unknown when these occurred in relation to dosing. With twice-nightly SXB, the US Prescribing Information was updated in 2014 to advise patients to stay in bed after they take the second dose of medication, with the recognition from postmarketing reports that falls have been reported when patients rise from bed [16, 42]. It is possible that ON-SXB may be less likely to cause these issues given the lower peak plasma concentration, the lower *C*_8h_, and once-nightly dosing.

Among the available narcolepsy treatments, only SXB has demonstrated improvement on nocturnal symptoms. While improvements in DNS have been reported post hoc with the immediate-release twice-nightly SXB therapy [30, 43], there is a bifurcation of the PSG data owing to the second, middle-of-the-night dose [30]. Additional analyses and publications are planned to understand if ON-SXB approximates a more “normal” sleep architecture, with a predominance of SWS early in the night and lighter stages of sleep closer to awakening.

ON-SXB achieved efficacy and safety compared to placebo at all doses evaluated (9, 7.5, and 6 g) for the three coprimary endpoints with a single nighttime dose, thus obviating the need for patients to disrupt their sleep by awakening in the middle of the night for a second dose that is required with conventional, immediate-release formulations of SXB. Clinically meaningful improvement was observed with the 6-g dose; moreover, this trial demonstrates for the first time that SXB, given as the once-nightly formulation, is effective at the 7.5-g dose. Adverse reactions were consistent with the known side effect profile of SXB and were generally mild or moderate. ON-SXB is under review at the FDA for the treatment of EDS and cataplexy in adults with narcolepsy with a Prescription Drug User Fee Act date of October 15, 2021 [44]. If approved, ON-SXB may represent a major advance for patients experiencing the burdensome symptoms of narcolepsy and for physicians who manage their patients with this chronic, debilitating sleep disorder.

## Data Availability

The data underlying this article will be shared on reasonable request to the corresponding author.
